# Motivation and response rates in bronchoscopy studies

**DOI:** 10.1186/s40248-019-0178-3

**Published:** 2019-05-02

**Authors:** Einar M. H. Martinsen, Tomas M. L. Eagan, Elise O. Leiten, Eli Nordeide, Per S. Bakke, Sverre Lehmann, Rune Nielsen

**Affiliations:** 1 Department of Clinical Science, University of Bergen, N-5021 Bergen, Norway; 2 Department of Thoracic Medicine, Haukeland University Hospital, N-5021 Bergen, Norway

**Keywords:** COPD, Clinical research, Motivation, Response rate, Non-response

## Abstract

**Background:**

Bronchoscopy is frequently used to sample the lower airways in lung microbiome studies. Despite being a safe procedure, it is associated with discomfort that may result in reservations regarding participation in research bronchoscopy studies. Information on participation in research bronchoscopy studies is limited. We report response rates, reasons for non-response, motivation for participation, and predictors of participation in a large-scale single-centre bronchoscopy study (“MicroCOPD”).

**Methods:**

Two hundred forty-nine participants underwent at least one bronchoscopy in addition to being examined by a physician, having lung function tested, and being offered a CT scan of the heart and lungs (subjects > 40 years). Each participant was asked an open question regarding motivation. Non-response reasons were gathered, and response rates were calculated.

**Results:**

The study had a response rate just above 50%, and men had a significantly higher response rate than women (56.5% vs. 44.8%, *p* = 0.01). Procedural fear was the most common non-response reason. Most participants participated due to perceived personal benefit, but a large proportion did also participate to help others and contribute to science. Men were less likely to give exclusive altruistic motives, whereas subjects with asthma were more likely to report exclusive personal benefit as main motive.

**Conclusion:**

Response rates of about 50% in bronchoscopy studies make large bronchoscopy studies feasible, but the fact that participants are motivated by their own health status places a large responsibility on the investigators regarding the accuracy of the provided study information.

**Electronic supplementary material:**

The online version of this article (10.1186/s40248-019-0178-3) contains supplementary material, which is available to authorized users.

## Introduction

Although primarily employed as a diagnostic tool, bronchoscopy is useful in studies on airway inflammation, bronchial remodelling, and the airway microbiota. Studies on airway microbiota have so far been relatively low-powered [1–3], and future studies will depend on larger samples. Even if bronchoscopy is associated with a low complication rate [4], some discomfort is inevitable, and potential participants may therefore have reservations [5]. Knowledge on motives for participation and response rates in bronchoscopy studies have the potential to optimise the recruitment process.

However, there are few studies providing reliable response rates and motives for participation in bronchoscopy studies. A literature review on research bronchoscopy studies included seven relevant studies, and found personal benefit and altruistic reasons to be the most important participation motives, whereas fear of the bronchoscopy was reported as a participation barrier [6]. Response rates from the seven studies varied from 3 to 73%, and no study examined participation among subjects with chronic obstructive pulmonary disease (COPD) in particular [6].

The Bergen COPD Microbiome study (“MicroCOPD”) is a large single-centre study of the airway microbiota, with bronchoscopic sampling of all participants. Data was collected at the Department of Thoracic Medicine, Haukeland University Hospital in Bergen, Norway, between April 2013 and June 2015. The main objective in the MicroCOPD study was to examine and compare airway microbiota from subjects without COPD (controls) and subjects with COPD. Some subjects with asthma were also included. The current paper reports response rates, reasons for non-response, motivation for participation, and predictors of participation in the MicroCOPD study.

## Methods

### Study design and population

The MicroCOPD study was a single-centre prospective, observational study carried out in Bergen, Western Norway. The study design has been described previously [7]. A pilot study of eight subjects with COPD was conducted in 2012 for protocol improvement. Participants from the pilot and main study were included in the current analyses. The main study included its first participant on April 11^th^, 2013, with the final study bronchoscopy performed June 5^th^, 2015. The study was conducted in accordance with the declaration of Helsinki and guidelines for good clinical practice. The regional committee of medical ethics approved the project (project number 2011/1307), and all participants provided informed written consent.

Controls and subjects with COPD or asthma were mainly recruited among participants of two previous studies performed by our research groups; the GeneCOPD study from 2003 to 2004 [8] and the Bergen COPD cohort study from 2006 to 2009 [9–12]. In addition, 6 subjects were recruited from outpatient clinics, and 8 subjects were recruited by their own initiative through attention from local media and hospital staff.

All subjects from the two previous studies who still lived in Bergen or the closest surrounding municipalities were eligible for participation. Potential participants were screened by an interview performed by a study physician regarding exclusion criteria for bronchoscopy before giving informed consent. We did not include subjects with increased bleeding risk, subjects with unstable cardiac conditions, or subjects with hypercapnia or hypoxaemia when receiving oxygen supplement [7]. Elderly subjects judged frail by the study physician were excluded. Participation was postponed for subjects that had used antibiotics or oral corticosteroids in the last 14 days, as well as subjects with symptoms of acute exacerbation of COPD.

### Data collection

Subjects that declined participation at the screening interview were asked about their non-response reason. Participants attended the outpatient clinic over one or two days depending on the availability of computed tomography (CT) scanning. A pulmonary and coronary CT scan was offered as part of a concurrent study, and this would be performed prior to bronchoscopy if the participants were scheduled for both procedures. At the day of bronchoscopy, prior to the procedure, participants underwent a structured interview regarding their medical history, respiratory symptoms, smoking habits, medication use, motivation, and exacerbation frequency if they had obstructive lung disease. An open question on motivation was first included in the study questionnaire from the fifth pilot patient, asked immediately prior to the procedure. Additionally, post-bronchodilator spirometry was performed and blood samples were collected.

Diagnoses of COPD and asthma were evaluated by the study physician, based on medical history, symptoms, pulmonary CT scan, and post-bronchodilator spirometry [13, 14]. Controls were judged to have no sign of airway or lung disease, based on the same information. After all participants were included, a panel of three physicians evaluated the diagnoses for a quality control regarding possible misclassification between controls and subjects with COPD or asthma.

The bronchoscopy procedure was explained in detail to each participant by the study physician immediately prior to the procedure. The procedure was performed with the participant in the supine position, with the option of light sedation (alfentanil, potentially combined with midazolam). Samples were gathered by sterile brushes and bronchoalveolar lavage (BAL) after application of a local anaesthetic agent. Additionally, gathering of bronchial biopsies began in May 2014. The details of bronchoscopic sampling have been previously published [7]. The average length of the bronchoscopy procedures was 15 min, including bronchoscopies with bronchial biopsies.

### Outcomes

Responders were subjects who accepted the invitation and underwent a bronchoscopy. Non-responders were subjects who did not undergo a bronchoscopy. Late non-responders were subjects who reconsidered an initial decision to participate, or when bronchoscopies were terminated before sampling due to participant discomfort. All non-responders were asked about their reason to decline participation. The response rate was defined as the number of bronchoscopies performed divided by the number of invited subjects.

Participation motives were collected from an open question before bronchoscopy started: “Why did you wish to take part in this project?”. Participants could provide more than one motive for participation, thus the overall numbers of motives exceeded the numbers of participants. At the time of analysis we initially merged the unique motives into 16 more principal motives, and then further classified these into three main groups: 1) *Altruism* was motivation by a wish to help others or a wish to continue participation from previous studies, as well as desire to contribute to science. 2) *Personal benefit* was motivation by a wish to somehow improve own health by participating in the project. 3) *Obligation* was a subjective feeling of being bound to participate. Subjects without specific reasons were labelled missing. We constructed binary variables “exclusive altruism” and “exclusive personal benefit” by coding them as ‘1’ if the participant only gave altruism or personal benefit as main motive, respectively. Participants stating both altruism and personal benefit, or altruism/personal benefit and obligation, were coded ‘0’ on these variables.

### Statistical analyses

All analyses were performed using Stata version 14 [15]. Response rates were stratified by sex and study category (control/obstructive lung disease). Chi-square test or Fisher’s exact test was used to compare frequencies of non-response reasons.

Bivariate analyses of responders and non-responders, as well as initial and late non-responders, were performed using parametric (t-test) and non-parametric tests (chi-square test or Fisher’s exact test), when judged appropriate. Bivariate logistic regression models were fitted with “exclusive altruism” or “exclusive personal benefit” as outcome. Covariates with *p* less than 0.20 before adjustment were included in multivariate models. In the logistic regression, age and FEV_1_ were treated as continuous variables, but divided by 10 to provide ratios for an increase of 10 units. Smoking habits were grouped according to current smoking status (never-, ex-, current-smokers), and we calculated number of pack/years (cigarettes per day divided by 20, multiplied by years smoking). Never-smokers and ex-smokers were merged into one category in the logistic regression analysis. Lung function was analysed using the percentage of predicted values of FEV_1_ and FVC, as well as the FEV_1_/FVC-ratio. Dyspnoea was classified according to the modified Medical Research Council (mMRC) dyspnoea scale [16].

## Results

### Flow chart (Fig. 1)

In total, 2,205 subjects from the two previous COPD cohorts were considered potential participants for the MicroCOPD study. 1,743 were ineligible, mainly due to death or that the MicroCOPD inclusion period ended (see Additional file 1: Table S1 for details). The total number of invited individuals for bronchoscopy was 462, of whom 323 subjects accepted the invitation. 85 subjects reconsidered their decision to participate, and further three bronchoscopies were terminated before sampling due to participant discomfort.

**Fig. 1:**
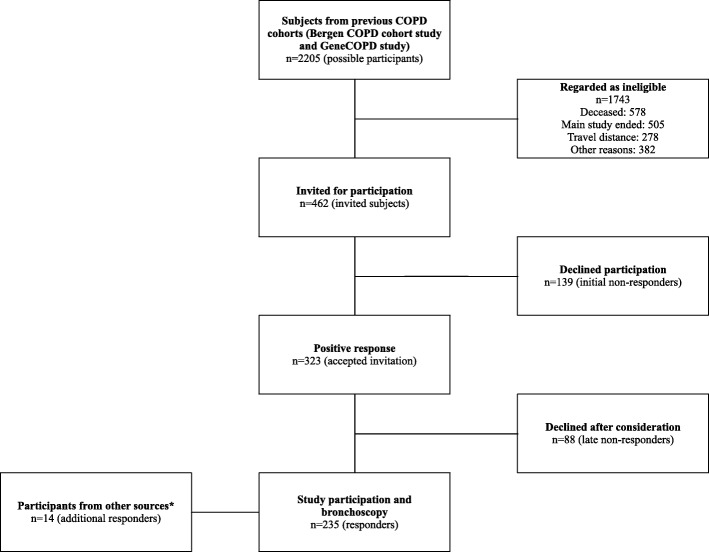
Flow chart of an observational research bronchoscopy study. * Local media, hospital staff, and outpatient clinics were regarded as other sources

### Response rates

Since the denominator of the response rate for subjects recruited from outpatient clinics, local media, and hospital staff was unknown, these 14 subjects were excluded from the response-rate analyses. Final response rate for the main study was 50.9%. The response rates in women and men were 44.8% (100/223) and 56.5% (135/239), respectively (*p* = 0.01). No significant difference in attendance was seen between subjects without obstructive lung disease and subjects with COPD or asthma.

### Demographics of responders and non-responders (Table 1)

There was no significant difference in age between responders and non-responders or between early or late responders. Whereas responders and initial non-responders did not differ by study category, there was a larger number of patients among the late non-responders compared with initial non-responders.

**Table 1:** Demographics of responders and non-responders in an observational research bronchoscopy study

Variable	Responders *n* = 235	Non-responders		*p*-*	p**
		Initial, *n* = 139	Late, *n* = 88		
Age (SD)	66.9 (7.6)	67.9 (8.0)	67.4 (7.5)	0.3	0.6
Sex				0.01	0.5
Women (%)	42.6	56.1	51.1		
Men (%)	57.4	43.9	48.9		
Study category				0.2	0.06
Controls (%)	43.0	41.7	29.6		
Obstructive lung disease (%)	57.0	58.3	70.4		

*Difference between responders and all non-responders

**Difference between initial and late non-responders

### Non-response reasons (Table 2)

Most initial non-responders stated that they feared the discomfort of a bronchoscopy (23.7%), and together with unspecific fear and worries related to study participation this accounted for 40.2% of all the initial non-response. The percentage of worries and fears in late non-responders was more than twice as high (*p* < 0.01). Among the initial non-responders there was a higher expression of study fatigue (10.1% vs. 2.3%, *p* = 0.03). A considerable number of the non-responders felt that their own health prevented participation (17.3% in initial non-responders, and 26.1% in late non-responders), and most so in subjects with obstructive lung diseases (*p* < 0.01).

**Table 2:** Self-reported non response-reasons in an observational research bronchoscopy study

Reasons	Non-responders			Study category		
	Initial, *n* = 139 Frequency (%)	Late, *n* = 88 Frequency (%)	*p*	Controls, *n* = 84 Frequency (%)	OLD, *n* = 143 Frequency (%)	*p*
Discomfort	33 (23.7)	16 (18.2)	0.3	19 (22.6)	30 (21.0)	0.2
Unspecified worries/fear concerning participation	23 (16.5)	30 (34.1)	< 0.01	19 (22.6)	34 (23.8)	0.08
Disease/health issues	24 (17.3)	23 (26.1)	0.1	8 (9.5)	39 (27.3)	< 0.01
Study fatigue	14 (10.1)	2 (2.3)	0.03	5 (6.0)	11 (7.7)	0.2
Time constraint	5 (3.6)	5 (5.7)	0.5	7 (8.3)	3 (2.1)	0.2
Practical^a^	5 (3.6)	6 (6.8)	0.3	5 (6.0)	6 (4.2)	0.9
Not satisfied with previous study participation	2 (1.4)	0	0.5	0	2 (1.4)	0.5
Feeling too old	2 (1.4)	0	0.5	2 (2.4)	0	0.2
Refuse to specify	1 (0.7)	0	1.0	1 (1.2)	0	0.5
Personal reason	0	3 (3.4)	0.06	1 (1.2)	2 (1.4)	1.0
Not specified	30 (21.6)	3 (3.4)	< 0.01	17 (20.2)	16 (11.2)	0.7

*OLD* obstructive lung disease

^a^Practical reflects practical issues for researcher or patient

### Detailed demographics of responders (Table 3)

The majority of responders were ex-smokers (68.3%), and a minority were never-smokers (9.6%). Age and sex were not significantly different between the controls and subjects with COPD. However, there were more ex-smokers and higher number of pack/years, less education, fewer married, more drug use, more comorbidities, as well as higher symptom burden and lower lung function among the subjects with COPD (*p* ≤ 0.01, tests not shown).

**Table 3:** Demographics of participants in an observational research bronchoscopy study

Variable	All, *n* = 249	Control, *n* = 103	COPD, *n* = 130	Asthma, *n* = 16
Age (SD)	66.3 (8.3)	65.3 (8.6)	67.2 (7.3)	65.5 (12.6)
Sex (men)	143 (57.4)	60 (58.3)	76 (58.5)	7 (43.8)
Number of medications (SD)	3.8 (3.2)	1.8 (1.7)	5.4 (3.3)	3.6 (2.4)
Number of comorbidities (SD)	1.1 (1.1)	0.8 (1.0)	1.4 (1.2)	0.8 (0.9)
FEV_1_, % of predicted (SD)	78.3 (28.2)	103.9 (12.3)	56.5 (19.2)	90.7 (13.3)
FVC, % of predicted (SD)	102.7 (18.7)	111.7 (13.5)	95.0 (19.2)	107.5 (16.5)
FEV_1_/FVC-ratio (SD)	0.6 (0.2)	0.7 (0.1)	0.5 (0.1)	0.7 (0.1)
Pack/years (SD)^a^	28.6 (20.1)	21.6 (16.9)	33.7 (20.5)	20.9 (22.0)
Smoking status (%)				
Daily	55 (22.1)	25 (24.3)	30 (23.1)	0 (0.0)
Ex-smokers	170 (68.3)	59 (57.3)	99 (76.2)	12 (75.0)
Never	24 (9.6)	19 (18.5)	1 (0.8)	4 (25.0)
Marital status (%)^a^				
Married/partner	157 (64.3)	79 (77.5)	72 (57.1)	6 (37.5)
Widowed	22 (9.0)	3 (2.9)	16 (12.7)	3 (18.8)
Cohabitant	20 (8.2)	5 (4.9)	13 (10.3)	2 (12.5)
Divorced, lives alone	33 (13.5)	11 (10.8)	17 (13.5)	5 (31.3)
Single	12 (4.9)	4 (3.9)	8 (6.4)	0 (0.0)
Education (%)^a^				
Primary school	48 (19.8)	12 (11.8)	35 (27.8)	1 (6.7)
Upper secondary/high school	125 (51.4)	52 (51.0)	67 (53.2)	6 (40.0)
3 years or more of higher education	70 (28.8)	38 (37.3)	24 (19.1)	8 (53.3)
mMRC Grade 2 and higher (%)				
Grade 2 level ground	30 (54.6)	3 (100)	27 (52.9)	0 (0.0)
Grade 3100 m	18 (32.7)	0 (0.0)	17 (33.3)	1 (100)
Grade 4 resting dyspnoea	7 (12.7)	0 (0.0)	7 (13.7)	0 (0.0)

*FEV*_*1*_ Forced Expiratory Volume in 1 Second*, FVC* Forced Vital Capacity, and *mMRC* modified Medical Research Council dyspnoea scale

^a^Due to some missing information, the sum of participants is not 249

### Motivation (Table 4)

Personal health benefit was the most common stated *principal motive* for participation (49.0%), followed by contribution to science (39.2%). 39 subjects (15.9%) also mentioned helping others as motivation. After merging into broader categories, primarily altruism was the *main motive* stated by most participants (67.3%), while 52.2% gave motives considered to be of personal benefit. Only 2.0% participated out of a sense of obligation.

**Table 4:** Motives reported by the 245^a^ participants who gave motives in an observational research bronchoscopy study

Motives	n	Percentage
*Primarily altruism* ^b^	*165*	*67.3*
Previous participation	23	9.4
Contribute to science	96	39.2
Help others	39	15.9
Give back (for previous participation)	7	2.9
Generally positive (to examination or participation and “yes-human”)	6	2.4
Social responsibility	3	1.2
COPD in family/among friends (including risk of COPD in family)	19	7.8
Available time	3	1.2
*Primarily personal benefit* ^b^	*128*	*52.2*
Personal health benefit	120	49.0
Experience the discomfort of bronchoscopy	1	0.4
Challenge	1	0.4
Curiosity	14	5.7
Fun	1	0.4
*Primarily obligation* ^b^	*5*	*2.0*
Acquaintance (in study, working with and was connected to the study or asked by)	4	1.6
Trust in authority/research	1	0.4
Missing	20	8.2

^a^Participation was not part of the questionnaire for the first four participants

^b^Unique motives are categorised into three main motives (in italic) by merging the unique motives listed below the main motive. The frequency (n) of main motives is not equal to the sum of each principal motive because a subject stating both "personal health benefit" and "challenge" would result in two observations in principal motives, but just one after merging

Frequencies on motives were also stratified by participant group, i.e. control, COPD, and asthma (see Additional file 1: Table S2 for details).

**Fig. 2:**
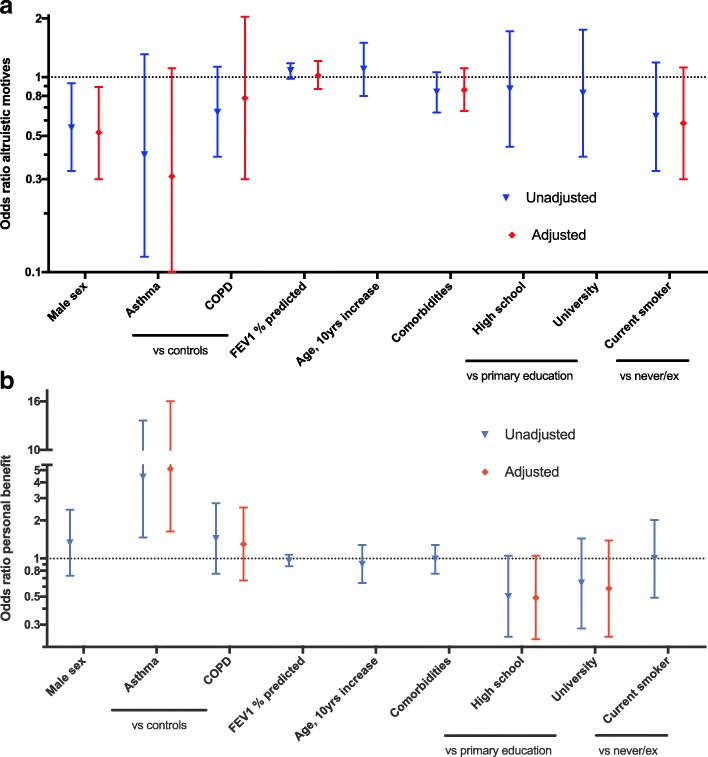
Logistic regression on **a**) exclusive altruism- and **b**) exclusive personal benefit-variables in an observational research bronchoscopy study

Men were less likely to state altruism as their main motive for participation (Fig. 2a, odds ratio (OR) 0.6, 95% confidence interval (0.3, 0.9)). This effect was more pronounced in the adjusted model, OR = 0.5 (0.3, 0.9). More subjects with asthma stated personal benefit motives than controls, unadjusted OR = 4.4 (1.5, 13.3), adjusted OR = 5.1 (1.6, 16.0) (Fig. 2b). No significant effect was observed by FEV_1_ in percentage of predicted, age, number of comorbidities, education, or smoking status.

## Discussion

We have reported response rates, non-response reasons, and motives for participation in a large single-centre bronchoscopy study. Response rates were about 50%, and did not differ between controls and subjects with COPD or asthma. The main reasons for non-response were fear of discomfort from the bronchoscopic procedure, and a subjective feeling of being diseased or too bothered from health issues to participate, especially among subjects with COPD or asthma. Participants were most frequently motivated by altruistic motives, but less so for men.

Given the invasiveness of the involved procedures, a response rate of 50% is not remarkably low. Little data exists on participation in research bronchoscopy studies [6]. Neither of seven Norwegian respiratory health surveys studies between 1965 and 1999 included a bronchoscopy, but baseline response rates varied from 68 to 90% [17]. Once attending, only 5% of attendants did not complete their participation [17]. In the current study, 27.2% (88/323) reconsidered their decision, suggesting higher rates of reconsideration with more invasive procedures.

A trend towards lower participation rates has been observed in Norwegian studies over time [17, 18]. This trend could at worst lead to selection bias and compromised external validity. In general, young, single men living in urban areas are the least likely to participate in social science surveys, while older women are the most willing [18]. In the current study, more men than women were responders. Experiences from clinical work suggest that women worry more about clinical procedures, and this could serve as a possible explanation for the observed difference. Additionally, more men were motivated by perceived personal benefit. If this observation stands true, one could speculate that male motivation is more easily satisfied by participation in a clinical study involving an actual diagnostic procedure, than participation in a questionnaire study. We observed no difference in mean age between responders and non-responders, but younger individuals were omitted from the current study, and frail elders were excluded. In another Norwegian study on respiratory health, non-response was related to lower age, rural habitation, and smoking habits [19]. Response rates from the current study will help researchers scale the number of invited subjects, aiming to recruit a sufficient number of participants, in order to avoid type II errors. These numbers can also be of value when investigators seek funding and ethics approval, providing precise information regarding the inclusion process.

Knowledge of reasons for non-response could guide researchers to provide precise information regarding the procedure during recruitment, which in turn might influence the willingness to participate. Observed difference in worries/fear between initial and late non-responders suggests that participants become frightened during the waiting time. Information on relevant discomfort should always be disclosed at first contact to avoid unreasonably procedural fear, and unnecessary waiting time before scheduled procedures should be avoided, both for research and clinical purposes. This will reduce costs and planning of non-performed procedures.

In agreement with the literature, we could categorise motives for participation into three groups, namely *personal benefit, altruism,* and *obligation* [6], although the review stated *obedience to the authority of the researchers* as an own group. Only one subject claimed trust in authority/research to be of importance in the current study. This discrepancy with previous studies might reflect both cultural differences and differences in health care organisation. Furthermore, patients are increasingly making their own health decisions [20], which might have changed the view of physicians as authorities.

We observed that women expressed more altruistic motives than men. In concordance with the current study, a meta-analysis on altruism and gender by Rand et al. showed women to be more intuitively altruistic, and men to be more selfish both intuitively and after consideration [21]. Our observation that subjects with asthma tend to report personal benefit needs to be interpreted with some caution. There were few subjects with asthma in our study, and they were recruited in a non-controlled manner.

Our results indicate that providing information on future implications of research can promote participation by appealing to a desire to contribute to science and future health care. Emphasising potential health benefits of study participation would probably have an even greater effect, but this warrants caution. Screening effects of bronchoscopy are not known. Also, there is a small complication risk associated with the procedure [4]. Participants were offered participation also in a concurrent study wherein a CT scan was offered, however participation in either study was not dependent on participation in the other. Thus, no exclusive, immediate benefit was received for the participants in the MicroCOPD study. Even though this was clearly stated in the written consent, almost half of the final participants stated personal benefit as an important motive. Participants’ expectation of perceived health benefit from participation is well known from the literature, even though no such benefit should be expected [5, 22], also where this is clearly stated by the research team [23]. Thus, we believe that participants´ perceived personal benefit in observational studies should be examined more thoroughly in future studies.

The MicroCOPD study is, to our knowledge, the largest single-centre lung microbiome study performed to date. We had extensive demographics on responders, and reliable results on motivation and non-response reasons. Some potential weaknesses deserve mentioning. Firstly, due to ethical and practical reasons, demographics on non-responders were sparse, and a considerable proportion of non-responders did not give any reason for their decline. Secondly, albeit a large study, the heterogeneity of the participants may have obscured the finding of important predictors of participation and motivation. Thirdly, an in-depth interview could have provided more insight into the details of non-response and motivation. Finally, most participants had already shown a willingness to take part in previous studies. Hence, they might be more prone to take part than a general population, generating some degree of selection bias. On the other hand, “study fatigue” might have lowered the participation rate in the current study.

## Conclusions

The response rate for research bronchoscopy in our study was 50%, and did not differ between controls and subjects with COPD or asthma. Non-responders refused participation mainly due to procedural fear. In contrast, responders were driven by perceived personal benefit, but a large proportion did also participate to help others and contribute to science. Our findings underline the importance of providing comprehensive information about the procedures. This might serve to avoid refusal on a possible misunderstood risk assessment, and to secure inclusion of a sufficient number of well-informed participants.

## Additional file

Additional file 1: **Table S1.** Reasons for ineligibility in an observational research bronchoscopy study, *n* = 1743. **Table S2.** Motives reported by the 245^a^ participants who gave motives in an observational research bronchoscopy study stratified by participant group. (PDF 96 kb)

## Abbreviations

“MicroCOPD”: The Bergen COPD Microbiome study
BAL: Bronchoalveolar lavage
COPD: Chronic obstructive pulmonary disease
CT: Computed tomography
OLD: Obstructive lung disease
