# Supplementary material for: Motivation and response rates in bronchoscopy studies
Source: Multidiscip Respir Med. 2019 May 2;14:14. doi: 10.1186/s40248-019-0178-3 (PMC6495518; doi:10.1186/s40248-019-0178-3)
Supplement: Supplementary file 1 — Table S1. Reasons for ineligibility in an observational research bronchoscopy study, n = 1743. Table S2. Motives reported by the 245a participants who gave motives in an observational research bronchoscopy study stratified by participant group. (PDF 96 kb) [file 40248_2019_178_MOESM1_ESM.pdf]

Additional material for "Motivation and response rates in bronchoscopy studies"

Table S1 Reasons for ineligibility in an observational research bronchoscopy study, n=1743

Reasons	n	Percentage
Travel distance	278	16.0
Age	48	2.8
Contact	32	1.8
Withdrawn from previous study	165	9.5
Moved from county	42	2.4
Main study ended	505	29.0
Deceased	578	33.2
Disease	26	1.5
Contraindicated	68	3.9
Not spirometric COPD	1	0.06

Table S2 Motives reported by the 245[a] participants who gave motives in an observational research bronchoscopy study stratified by participant group

Motives	All, n=245 (%)	Control, n=103 (%)	COPD, n=126 (%)	Asthma, n=16 (%)
Primarily altruism[b]	*165 (67.3)*	*76 (73.8)*	*81 (64.3)*	*8 (50)*
Previous participation	23 (9.4)	13 (12.6)	10 (7.9)	0
Contribute to science	96 (39.2)	43 (41.7)	46 (36.5)	7 (43.8)
Help others	39 (15.9)	12 (11.7)	25 (19.8)	2 (12.5)
Give back (for previous participation)	7 (2.9)	2 (1.9)	5 (4.0)	0
Generally positive (to examination or participation and "yes-human")	6 (2.4)	4 (3.9)	2 (1.6)	0
Social responsibility	3 (1.2)	3 (2.9)	0	0
COPD in family/among friends (including risk of COPD in family)	19 (7.8)	13 (12.6)	6 (4.8)	0
Available time	3 (1.2)	2 (1.9)	1 (0.8)	0
Primarily personal benefit[b]	*128 (52.2)*	*49 (47.6)*	*67 (53.2)*	*12 (75)*
Personal health benefit	120 (49.0)	46 (44.7)	62 (49.2)	12 (75)
Experience the discomfort of bronchoscopy	1 (0.4)	1 (1.0)	0	0
Challenge	1 (0.4)	0	1 (0.8)	0
Curiosity	14 (5.7)	8 (7.8)	6 (4.8)	0
Fun	1 (0.4)	0	1 (0.8)	0
Primarily obligation[b]	*5 (2.0)*	*3 (2.9)*	*2 (1.6)*	*0*
Acquaintance (in study, working with and was connected to the study or asked by)	4 (1.6)	2 (1.9)	2 (1.6)	0
Trust in authority/research	1 (0.4)	1 (1.0)	0	0
Missing	20 (8.2)	7 (6.8)	13 (10.3)	0

[a]Participation was not part of the questionnaire for the first four participants.

[b]Unique motives are categorised into three main motives (in italic) by merging the unique motives listed below the main motive. The frequency (n) of main motives is not equal to the sum of each principal motive because a subject stating both "personal health benefit" and "challenge" would result in two observations in principal motives, but just one after merging.
